# The catalytic inhibitor epacadostat can affect the non-enzymatic function of IDO1

**DOI:** 10.3389/fimmu.2023.1134551

**Published:** 2023-04-14

**Authors:** Eleonora Panfili, Giada Mondanelli, Ciriana Orabona, Marco Gargaro, Claudia Volpi, Maria Laura Belladonna, Sofia Rossini, Chiara Suvieri, Maria Teresa Pallotta

**Affiliations:** Department of Medicine and Surgery, University of Perugia, Perugia, Italy

**Keywords:** indoleamine 2,3-dioxygenase 1 (IDO1), epacadostat, tryptophan, kynurenine, IDO1-mediated signaling

## Abstract

Indoleamine 2,3-dioxygenase 1 (IDO1) is a tryptophan metabolizing enzyme chronically activated in many cancer patients and its expression and activity correlate with a poor prognosis. In fact, it acts as an immune regulator and contributes to tumor-induced immunosuppression by determining tryptophan deprivation and producing immunosuppressive metabolites named kynurenines. These findings made IDO1 an attractive target for cancer immunotherapy and small-molecule inhibitors, such as epacadostat, have been developed to block its enzymatic activity. Although epacadostat was effective in preclinical models and in early phase trials, it gave negative results in a metastatic melanoma randomized phase III study to test the benefit of adding epacadostat to the reference pembrolizumab therapy. However, the reason for the epacadostat failure in this clinical trial has never been understood. Our data suggest that a possible explanation of epacadostat ineffectiveness may rely on the ability of this drug to enhance the other IDO1 immunoregulatory mechanism, involving intracellular signaling function. These findings open up a new perspective for IDO1 inhibitors developed as new anticancer drugs, which should be carefully evaluated for their ability to block not only the catalytic but also the signaling activity of IDO1.

## Introduction

Immune checkpoint blockade therapy was developed as a new therapeutic strategy with the aim to strengthen the anti-tumor immune response to eliminate tumor cells (1). This approach has been applied into an increasing number of solid malignancies and has significantly improved clinical outcomes in some of them (2).

Tumor cells could escape destruction by the immune system through multiple local immunosuppression mechanisms. Indoleamine 2,3-dioxygenase 1 (IDO1) is recognized as an immune checkpoint molecule that is expressed in 50% of human tumors and plays a major role in tumor escape mechanisms. It is a heme-containing enzyme that catalyzes the initial rate-limiting step in the degradation of the essential amino acid tryptophan. This enzymatic activity causes the production of L-kynurenine, which is the upstream metabolite along the so-called kynurenine pathway (3). IDO1 induces immunosuppressive responses in dendritic cells (DCs) by multiple mechanisms depending on its enzymatic and signaling functions. It causes tryptophan deprivation and the production of kynurenines, which act on IDO1^+^ DCs and T cells, switching them into regulatory cells that mediate T cell inhibition of proliferation, apoptosis, and differentiation (4, 5). These effects depend on the ability of L-kynurenine to bind and activate the aryl hydrocarbon receptor (AhR), which induces IDO1 expression and generates a self-amplification circuit for the maintenance or the new onset of a stably regulatory phenotype in DCs (6). IDO1 expression in tumor cells is associated with significantly worse clinical prognosis and reduced overall survival in many cancer types. Therefore, intense efforts have been to design and develop selective inhibitors of the catalytic activity of IDO1 as a goal to enhance anti-tumor immune responses (7). Epacadostat is a potent and selective IDO1 inhibitor, which has displayed the ability to restore anti-tumor T cell immunity and synergizes with immune checkpoint inhibitors in preclinical models and in early phase trials (8). Thus, a randomized phase III study (ECHO-301/KN-252) was launched in metastatic melanoma to test its synergistic therapeutic effects in combination with the anti-PD-1 monoclonal antibody pembrolizumab. However, epacadostat gave no benefit respect to the reference pembrolizumab therapy (9) and consequently, other trials have been suspended, canceled, or downsized. Several hypotheses were developed with the aim to understand the reason for this clinical failure, among them that epacadostat could affect the IDO1 non-enzymatic function (10).

We previously demonstrated that IDO1 also acts as a signal-transducing molecule *via* two Immunoreceptor Tyrosine-based Inhibitory Motifs (ITIMs) located in the small, non-enzymatic domain of the enzyme (11, 12). When the tyrosine residue within these motifs become phosphorylated, IDO1 associates with SHP’s family tyrosine phosphatases. Moreover, through its YxxM (‘x’ indicates any amino acid) phosphorylation motif, IDO1 can directly bind the class IA PI3K regulatory subunit. These events lead to IDO1 anchoring to early endosomes (EE) and the activation of the ITIM-mediated signaling pathway that confers a long-term immunoregulatory phenotype in conventional DCs (cDCs) and plasmacytoid DCs (pDCs) (13). The present study aims to investigate the effect of epacadostat on the IDO1 signaling-mediated function.

## Materials and methods

### Animals and *reagents*

Female wild-type C57BL/6 mice of 6-wks of age were purchased from Charles River Breeding Laboratories Italia (Milan, Italy). All animals were housed and fed under specific pathogen free conditions in the animal facility of the University of Perugia. All *in vivo* studies were compliant with National (Italian Animal Welfare Assurance A-3143-01) and Perugia University Animal Care and Use Committee guidelines. DMSO was purchased from Sigma-Aldrich (MO, USA). The H-2Db-restricted HY peptide (WMHHNMDLI) — containing the immunodominant epitope of male mouse specific minor transplantation antigen — was synthesized by BioFab Research (Rome, Italy). Epacadostat was purchased from Selleckchem (Huston, TX, USA).

### Cellular assays

Cells of P1.HTR, a highly transfectable clonal variant of mouse mastocytoma P815 (14), were transfected by electroporation with a plasmid construct coding for mouse wild type (wt) (P1.IDO1), or mutant IDO1 lacking the catalytic activity, namely H350A (P1.H350A), as described in (11, 15). The stable transfectant cell line was obtained by puromycin selection and cultured in Iscove’s Modified Dulbecco’s Medium (Gibco, Invitrogen CA, USA) supplemented with 10% FCS (Gibco, Invitrogen CA, USA), 1mM glutamine (Gibco, Invitrogen CA, USA) and penicillin/streptomycin (Gibco, Invitrogen CA, USA) at 37°C in a humidified 7% CO_2_ incubator. In every cellular assay, the cell line was used at a passage number not exceeding the 10^th^. Dose-response curve for extrapolating the IC_50_ (half-maximal inhibitory concentration) of epacadostat in P1.IDO1 cells was built in a cellular assay, as described in (16) by incubating cells with 2-fold serial dilutions of the compound. After the incubation, supernatants of cell cultures were recovered and the kynurenine concentration was detected by HPLC. The control was represented by cells incubated with an equivalent volume of DMSO, the vehicle in which epacadostat has been solubilized. Every cell assay was conducted in triplicate and repeated three times.

### Kynurenine measurements

Detection of kynurenine concentrations was performed by using a Perkin Elmer, series 200 HPLC instrument (MA, USA). A Kinetex^®^ C18 column (250×4.6 mm, 5 μm, 100 Å; Phenomenex, USA), maintained at the temperature of 25°C and pressure of 1800 PSI, was used. A sample volume of 300 μl was injected and eluted by a mobile phase containing 10mM NaH_2_PO_4_ pH 3.0 (99%) and methanol (1%) (Sigma-Aldrich, MO, USA), with a flow rate of 1 ml/min. Kynurenine was detected at 360 nm by an UV detector. The software TURBOCHROM 4 was used for evaluating the concentration of kynurenine in samples by mean of a calibration curve. The detection limit of the analysis was 0.05 μM.

### MTT 3-(4,5-Dimethylthiazol-2-yl)-2,5-Diphenyltetrazolium Bromide) assay

P1.HTR cells (25 × 10^3^ cells/well) were seeded into a flat-bottom 96-well plate and incubated overnight at 37°C with scalar concentrations of epacadostat or DMSO as a control. Then, medium containing MTT (Sigma Aldrich, St. Louis, MO, USA) was added and cells were incubated for 4 h at 37°C. Subsequently, solubilization buffer (SDS 10% in HCl 0.01 M) was added, the plate was incubated overnight at 37°C, and absorbance at 570 nm was measured using a UV/visible spectrophotometer (TECAN, Thermo Fisher Scientific, Waltham, MA, USA) to construct a graph of cell viability. The assay was performed in triplicate for each concentration.

### Fluorescence resonance energy transfer (FRET) analysis

Constructs used for FRET analysis were SHP-1-GFP (Ptpn6 Mouse Tagged ORF Clone, Origene) and IDO1-RFP (mouse *Ido1* were cloned in pTagRFP-N vector, Evrogen). Both constructs were confirmed by DNA sequencing. HeLa cells (4 x 10^5^ cells/96-mm dish) were plated on coverslips for 24 h and then co-transfected with SHP-1-GFP and IDO1-RFP plasmids DNA using Lipofectamine 3000 (Thermo Fisher Scientific), according to the manufacturer’s protocol. After 16 h, transfected cells were incubated for 4 h with epacadostat 1 μM or DMSO as control. Prior to the FRET imaging, cells were washed twice with PBS 1X, treated with PFA 4%, and then mounted with Prolong-DAPI (ThermoFisher). Samples were imaged using a Nikon inverted microscope (60X magnification) using a GFP/RFP FRET pair and processed using ImageJ software (National Institute of Health). Acquired fluorescent images were background-subtracted, and multiple regions of interest (ROIs) were selected for quantitative data analysis (~10–20 ROIs per cell, and 6–8 cells per condition were averaged). FRET efficiency values presented as a pseudo-color scale by ImageJ. Cells transfected with either SHP-1-GFP or IDO1-RFP were used to calculate the coefficient A (CoA) and B (CoB), that correct for the amount of acceptor bleedthrough in the absence of a donor and that of donor bleedthrough in the absence of an acceptor, respectively.FRET efficiency was calculated as follows:


FRET corr=DaFRET−(coB ×DdFRET)−(coA ×AaFRET)

$$\%\text{ Efficiency }=\frac{FRETcorr\;}{DdFRET\;}\;\times 100$$

### Immunoprecipitation and western blotting

For immunoprecipitation of IDO1, a rabbit monoclonal anti-mouse IDO1 antibody (CV152) obtained in our laboratory (11) was used. Antibodies used to analyze immunoprecipitated samples were SH-PTP1 (clone C-19 Cat# sc-287) and SH-PTP2 (clone B-1 Cat #sc-7384), both from Santa Cruz Biotechnology (TX, USA), and PI3K p85 (rabbit mAb clone 19H8 Cat #42575) from Cell Signaling Technology (MA, USA). Anti-Rab5 (rabbit mAb clone C8B1 Cat# 3547) were from Cell Signaling Technology (MA, USA).

### Phosphatase assay

P1.IDO1 and P1.H350A cells were pre-incubated for 4 h with epacadostat 1 μM or DMSO as control, before assessing the phosphatase activity associated with the IDO1 protein. For each condition, the IDO1 protein was immunoprecipitated from 6 x 10^6^ cells lysed in 400 μl of Lysis Buffer (50 mM Tris-HCl pH 7.5, 150 mM NaCl, 1% Nonidet P-40 and a cocktail of protease inhibitors). The IDO1 protein was immunoprecipitated overnight at 4°C by a specific monoclonal rabbit antibody raised in our lab, followed by addition of Protein G-Sepharose (Sigma-Aldrich, MO, USA) for 2 h. The phosphatase cactivity associated with immunoprecipitated IDO1 was assessed by a specific kit purchased from Promega (WI, USA), according to the manufacturer’s instructions.

### Endosome isolation

Endosomes were isolated using a Minute Endosome Isolation and Cell Fractionation Kit (Invent Biotechnologies, Inc., MN, USA) according to the manufacturer’s instructions. Briefly, 3×10^7^ cells were collected, washed with cold PBS and resuspended in buffer A. After incubation, the cell suspension was transferred to a filter cartridge and centrifuged twice. Next, the pellet was resuspended and centrifuged twice. After centrifugation, supernatant was transferred to a fresh tube, mixed with buffer B, incubated overnight at 4°C, and centrifuged. The pellet resulting from the centrifugation contains isolated endosomes, whereas the supernatant represents the cytosolic fraction. Both fractions were analyzed by western blotting.

### DC purification and skin test assay

All purification procedures and purities of splenic pDCs and CD8^−^ DCs (hereafter referred to as cDCs, for conventional DCs) were conducted as previously described in (17, 18). Briefly, splenic DCs were purified using CD11c MicroBeads (Miltenyi Biotec) in the presence of EDTA to disrupt DC-T cell complexes. Cells were >99% CD11c^+^, >99% MHC I-A^+^, >98% B7–2^+^,<0.1% CD3^+^, and appeared to consist of 90-95% CD8^-^, 5–10% CD8^+^, and 1–5% B220^+^ cells. DC populations were further fractionated according to CD8 expression to obtain purified CD8^+^ DCs and CD8^-^ DCs by means of CD8α MicroBeads (Miltenyi Biotec). After cell fractionation, the recovered CD8^-^ cells typically contained<0.5% contaminating CD8^+^ DCs, whereas the CD8^+^ fraction was made up of >95% CD8^+^ DCs. Less than 3% CD11c^+^ cells expressed the mPDCA-1 marker. A skin test assay was used for measurements of major histocompatibility complex class I-restricted delayed-type hypersensitivity (DTH) responses to the HY peptide in C57BL/6 female recipient mice (19).

For the antigen-specific immunization of female recipient mice was used the HY peptide because is a male histocompatibility antigen that causes rejection of male skin grafts by female recipients (20).

For *in vivo* immunization, 3×10^5^ peptide-loaded cDCs, combined with a minority fraction (5%) of peptide-loaded pDCs, were injected subcutaneously into recipient mice. After two weeks, mice were challenged intrafootpad with the HY peptide in the absence of DCs (experimental footpad) or with vehicle alone (control counterpart). Twenty-four hours later, mice were sacrificed, footpads were removed and the change in the weight of the peptide-injected footpad over that of the vehicle-injected counterpart (i.e., internal control) was measured. A significant increase in the mean weight of the experimental over the control footpads will then demonstrate and quantify any delayed type, antigen-specific hypersensitivity response. Representative pictures of the footpads from a positive and negative DTH response have been already published in (11). Results are represented as the mean weight ± SD of the experimental footpads over that of control, vehicle-injected counterparts. Measurements were performed in a blinded fashion. The minority cell fraction, constituted by pDCs, was left untreated or treated overnight with DMSO or epacadostat 1 μM.

### Real-time PCR analysis

Expression of *Ido1* and *Tgfb1* genes was analyzed by Real-Time PCR, as described [21] using the specific primers reported in (16). Real-time PCR assays were performed using Stratagene Mx3005P (Agilent Technologies, CA, USA). mRNA levels were normalized to the expression of the *Gapdh* housekeeping gene. The Ct number was measured using the MxPro-Mx3005P software (Agilent Technologies, CA, USA). Values were expressed as the ratio of *Gapdh* normalized transcript expression of compound treated-pDCs to *Gapdh* normalized transcript expression.

### Flow cytometry analysis

pDCs were harvested by centrifugation at 1400 r.p.m for 5 minutes, followed by surface staining for 30 minutes at 4°C in 1x PBS + 0.5% BSA and 2mM EDTA (MACS buffer). Subsequently, surface antigens were stained in MACS buffer for 30 minutes at 4°C. Samples were fixed by incubation in 1x PBS + 1% (v/v) paraformaldehyde. Cells were analyzed with FACS Fortessa and data was analyzed with FlowJo software (Tree Star).

### Statistical analysis

All analyses were performed using Prism version 8.0.1 (GraphPad Software). Data usually met normality and were analyzed by two-tailed unpaired Student’s t-test when two samples were under comparison or ANOVA followed by *post-hoc* Bonferroni’s test, when three or more samples were under comparison. A P value less than 0.05 was considered significant. Overall results, obtained by at least three replicates per experimental parameter, were shown as mean ± SD.

## Results

### Epacadostat promotes IDO1/SHPs association in tumor cells

**Figure 1:**
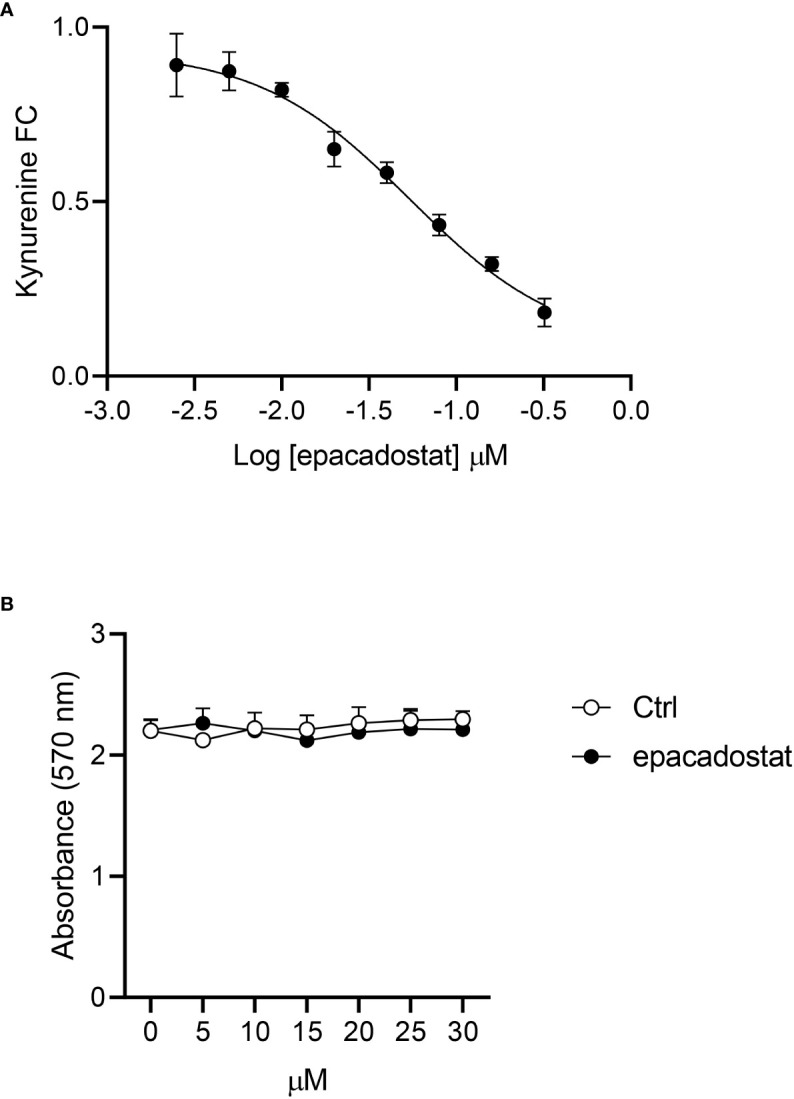
Epacadostat inhibits the IDO1 enzymatic activity in P1 tumor cells. **(A)** Dose–response curve of epacadostat used to calculate the IC_50_ value in P1.IDO1 cells. Results are the mean ± SD of the kynurenine fold change (FC) of three independent experiments, each performed in triplicate. **(B)** Cell viability percentage (%) of P1.IDO1 cells treated with epacadostat or the vehicle alone as a control (Ctrl). Results are the mean ± SD of three experiments, each conducted in triplicate.

Before investigating the effect of epacadostat on IDO1-mediated signaling, we tested the ability of this compound to inhibit the enzymatic activity of IDO1 in the mastocytoma cell line P1.HTR stably expressing the mouse IDO1 protein (P1.IDO1). By treating the cells with different concentration of the compound, we could confirm that epacadostat can inhibit the conversion of tryptophan into kynurenine also in P1.IDO1. The obtained concentration curve provided a cellular IC50 of 54.46 nM ± 11.18 nM, a value very similar to that reported in the literature (i.e. IC50 88 nM (21)) (**Figure 1A**). In order to exclude a cytotoxic effect of the compound, we first performed a MTT assay that measures cellular metabolic activity as an indicator of cell viability. By using seven 2-fold dilutions in the 1–30 μM concentration range, we did not observe any cytotoxic effect of epacadostat for all the concentrations tested (**Figure 1B** and **Supplementary Figure 1**).

**Figure 2:**
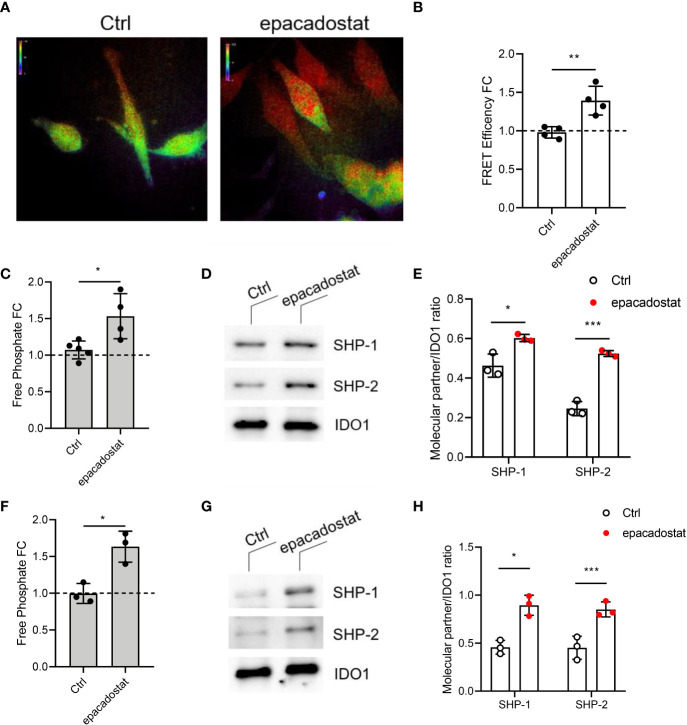
The IDO1 binding to tyrosine phosphatases SHPs is enhanced by epacadostat. **(A)** Representative FRET microscopy images of GFP/RFP emission in HeLa cells expressing IDO1-RFP and SHP-1-GFP fusion protein treated with epacadostat (1 μM) or the vehicle alone as a control (Ctrl). Pseudocolor scale, red corresponds to maximum interaction, while purple indicates the absence of proteins proximity. One experiment representative of two is shown. **(B)** FRET efficiency values are presented as fold change (FC) of cells treated with epacadostat (1 μM) relative to untreated counterparts (fold change = 1, dotted line). Data ± SD are the results of two independent experiments, each performed in triplicate. **(C)** Phosphatase activity produced by IDO1 immunoprecipitated from P1.IDO1 cells treated with epacadostat (1 μM) or the vehicle alone as a control (Ctrl). Levels of free phosphate (pmol) generated during the incubation of a tyrosine-phosphorylated peptide with the co-immunoprecipitated IDO1 proteins from each condition were presented as a fold change (FC) relative to untreated cells (dotted line). Results are representative of three independent experiments (mean ± S.D.), each performed in triplicate. **(D)** Immunoblot analysis of coimmunoprecipitates from P1.IDO1 cells treated as in **(C)**. One experiment representative of three is shown. **(E)** Quantitative analysis of three independent immunoblot experiments, one of which represented in **(D)**. Analysis of phosphatase activity **(F)**, immunoblot **(G)**, and quantitative analysis **(H)** conduced as in **(C–E)** in cells stably expressing the IDO1 mutant H350A. One experiment representative of three is shown. Data ± SD represent the ratio of co-immunoprecipitated partner protein over immunoprecipitated IDO1. Data in **(B, C, E, F, H)** were analyzed with Unpaired Student’s t-test. *p< 0.05, **p< 0.01, ***p< 0.001.

Whether epacadostat could affect also the non-enzymatic function of IDO1 was never investigated. Because the first event which activates the IDO1-mediated signaling pathway is the association with tyrosine phosphatases SHPs (11, 12, 18, 22), we examined whether this compound could have a role in the IDO1-SHPs binding. HeLa cells transiently co-expressing IDO1–RFP and SHP-1–GFP were treated with epacadostat or the vehicle alone to determine if the compound could affect the association between these two proteins by fluorescence resonance energy transfer (FRET) microscopy. We found that IDO1 interacts with SHP-1 (**Figure 2A**) and SHP-2 (**Supplementary Figure 2**) in HeLa cells in absence of any stimulus, consistently with previous reports in P1.IDO1 cells (11). Surprisingly, epacadostat enhanced the association between these proteins (**Figures 2A, B** and **Supplementary Figures 2A, B**). This finding was confirmed by the evaluation of the ability of IDO1 immunoprecipitates to remove a phosphate group from a tyrosine-phosphorylated substrate, as described (11, 13, 15). Specifically, we quantified the amount of free phosphate produced by IDO1 immunoprecipitated from P1.IDO1 cells untreated, treated with epacadostat or the vehicle as a control. As previously demonstrated (11), we observed a basal phosphatase activity associated with IDO1 immunoprecipitates obtained from P1.IDO1 cells. However, preconditioning of the cells with epacadostat for 4 h generated a significantly increased level of free phosphate, as compared to the control (**Figure 2C**). Western blot analysis of the coimmunoprecipitates and quantitative analysis of molecular partners confirmed that both the SHPs enhance their binding to IDO1 in the presence of epacadostat (**Figures 2D, E** and **Supplementary Figure 3**). Interestingly, by using our previously characterized IDO1 mutant lacking the histidine residue required for catalytic activity (i.e. H350A (11)), we found that IDO1 can bind the same epacadostat, which still improves the first event in the IDO1-signaling functions (**Figures 2F–H** and **Supplementary Figure 4**).

Collectively, these data indicate that in the mastocytoma tumor cell stably expressing IDO1, epacadostat can effectively inhibit the catalytic activity of this enzyme. However, it can also improve the ability of IDO1 to interact with SHP phosphatases, previously described as the first molecular event triggering the signaling function of IDO1 (11).

### Epacadostat affects the IDO1 intracellular trafficking

**Figure 3:**
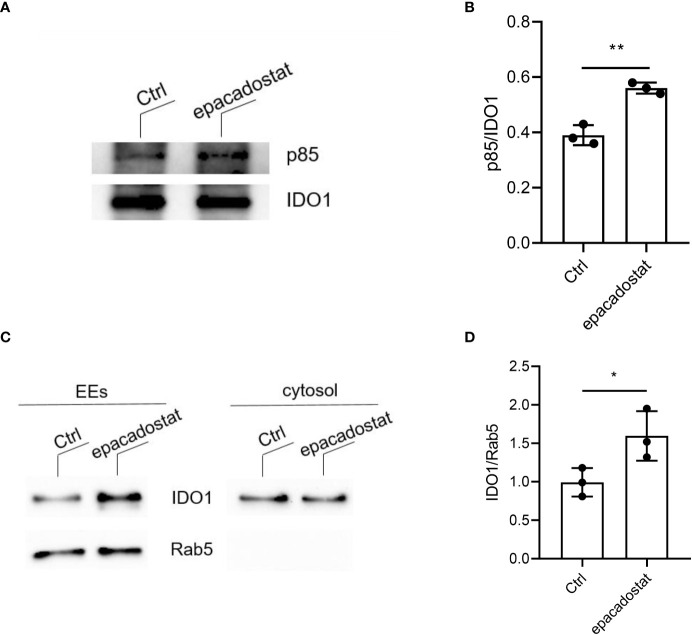
Epacadostat favors the IDO1 signaling localization in tumor transfectants. **(A)** Immunoprecipitation of IDO1 from WCL of P1.HTR IDO1 transfectants treated with epacadostat (1 μM) or the vehicle alone (Ctrl), and detection of IDO1 or its molecular partner p85 PI3K by sequential immunoblotting with specific antibodies. One experiment representative of three is shown. **(B)** Quantitative analysis of three independent immunoblot experiments, one of which represented in **(A)**. Data ± SD represent the ratio of co-immunoprecipitated p85 protein over immunoprecipitated IDO1. **(C)** Immunoblot analysis of endosomes (EEs) and cytosol isolated from epacadostat-treated P1.IDO1 cells. Cells incubated with the vehicle DMSO were used as a control (Ctrl). One experiment representative of three is shown. **(D)** Quantitative analysis of three independent immunoblot experiments, one of which represented in **(C)**. IDO1:Rab5 ratio was calculated by densitometric quantification of the specific bands detected in three independent experiments (mean ± S.D.). *p< 0.05 **p< 0.01 (Paired Student’s t- test).

In the literature, IDO1 is described as a cytoplasmic enzyme. However, we recently demonstrated that under conditions that favor the signaling rather than the catalytic activity of IDO1, the protein can shift from the cytosol to early endosomes (EEs) where it plays a signalling activity (12, 13). We thus investigated whether epacadostat may regulate IDO1 intracellular trafficking. A mandatory event for the anchoring of IDO1 to EEs is its direct interaction with class IA PI3Ks p85 *via* a YxxM motif (12, 13). Therefore, we co-immunoprecipitated IDO1 from P1.IDO1 cell lysates pretreated with epacadostat or the vehicle alone, as control. We found that the p85 subunit of PI3K efficiently co-precipitated with IDO1 and the association was enhanced in epacadostat-treated cells (**Figures 3A, B** and **Supplementary Figure 5**). To further investigate the effect of epacadostat on the cellular topology of IDO1, we isolated EEs from P1.IDO1 cells using a subcellular fractionation approach. In accordance with our previous data (13), the immunoblotting assay verified that in a tumor transfectant cell the IDO1 protein is not just confined to cytosol but can also associate with EEs. However, this intracellular localization of IDO1 was significantly favored by the presence of epacadostat (**Figures 3C, D** and **Supplementary Figure 6**).

As a whole, these data indicate that epacadostat promotes the molecular events and the intracellular trafficking required for IDO1-mediated signaling pathway, by increasing the interaction of IDO1 with PI3K and making it move toward the EEs.

### Epacadostat confers a tolerogenic phenotype on pDCs

**Figure 4:**
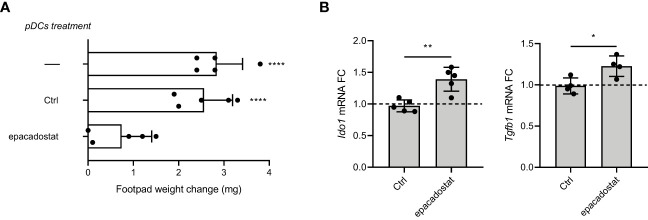
Epacadostat confers a tolerogenic phenotype on pDCs. **(A)** Skin test reactivity of mice sensitized with splenic HY‐pulsed immunostimulatory cDCs combined with a minority fraction (5%, indicated) of pDCs. pDCs were left untreated, treated with the vehicle DMSO (Ctrl) or stimulated with epacadostat 1 μM prior to be HY-pulsed together with cDC majority fraction and i.p. transferred into syngeneic C57BL/6 recipient female mice. Two weeks after the immunization, mice were assayed by footpad challenge with HY peptide. Skin test reactivity of the recipient mice (n=6 per group) to the eliciting peptide is represented as change in weight of treated footpad vs vehicle‐receiving counterpart. Data are reported as mean value ± S.D. of three experiments. **(B)** Real‐time PCR analysis of *Ido1* and *Tgfb1* transcripts. Data (means of three experiments using triplicate samples) represent the *Ido1* and *Tgfb1* transcript fold change in 1 μM epacadostat–treated relative to untreated pDCs, all normalized to the *Gapdh* expression. Dotted line, fold change = 1. Data are reported as mean value ± S.D. of three experiments. Data in **(A)** are presented as change in footpad weight and significance is referred to a Tukey’s multiple comparison test (n=6 mice/group for (n=3)) ****P< 0.0001. Data in **(B)** were analyzed with Unpaired Student’s t-test. *p< 0.05, **p< 0.01.

The IDO1 signaling functions were first described in pDCs and cDCs, immune cells characterized by a plastic phenotype prompt to respond to any environmental signal (11, 18). In particular, pDCs (**Supplementary Figure 7**) represent the most flexible DC subset and can shift from an immunostimulatory toward an immunosuppressive program by sensing the extracellular milieu (23). In order to analyze the effect of epacadostat on the immunoregulary function of pDCs, we investigated the *in vivo* priming ability of these antigen presenting cells after *in vitro* pre-conditioning with epacadostat. Specifically, we sensitized female mice with the H-2Db–restricted HY peptide presented by a combination of immunogenic cDCs and a minority fraction of pDCs preincubated *in vitro* overnight with epacadostat or DMSO, as control. Two weeks later, we evaluated the antigen-specific reactivity through an intrafootpad challenge of the HY antigen, according to an established protocol for measuring the induction of immune reactivity *versus* tolerance (19). As expected, sensitization of mice with a combination of immunogenic cDCs and vehicle-treated pDCs elicited a positive response against the HY antigen in mice. In contrast, epacadostat-pretreated pDCs abrogated the immunoadjuvant response elicited by cDCs, suggesting that this compound could induce in pDCs a tolerogenic phenotype (**Figure 4A** and **Supplementary Figures 8A, B**).

**Figure 5:**
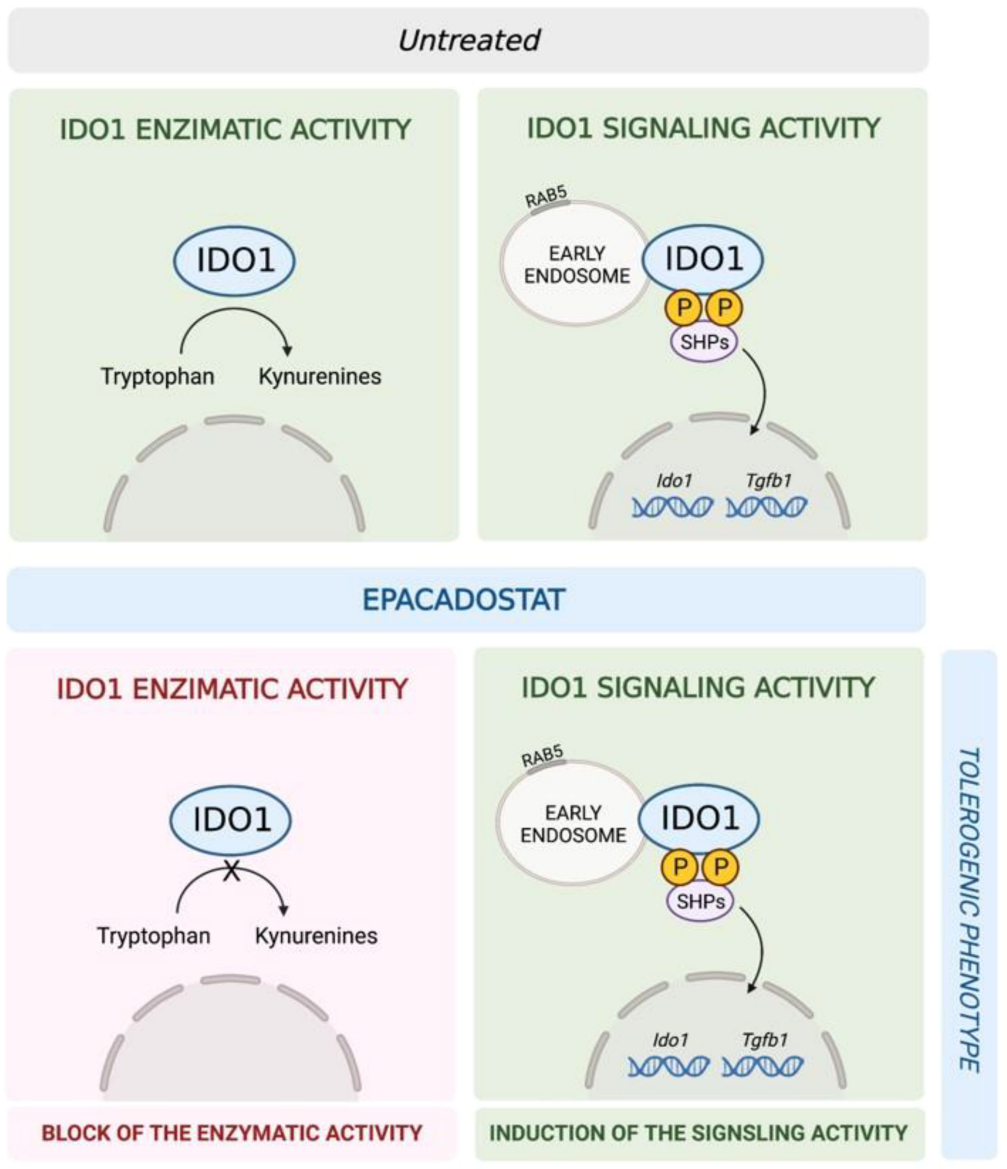
Schematic representation of the epacadostat effects on IDO1 in pDCs. In untreated pDCs (*upper panel*) IDO1 is capable to perform both the enzymatic and the signaling activity. Epacadostat (*lower panel*) inhibits the IDO1 enzymatic activity but enhances IDO1-mediated signaling events, thus resulting in the induction of a tolerogenic phenotype in pDCs.

We previously demonstrated that in a microenvironment dominated by the immunoregulatory cytokine TGF-β, the activated PI3K-IDO1-SHPs axis promotes the nuclear translocation of the noncanonical pathway of NF-κB, leading to the *de novo* expression of *Ido1* and *Tgfb1* genes. This mechanism establishes a positive immunoregulatory circuitry responsible for a long-term immunoregulatory phenotype of the DCs and pDCs (11–13, 18). Thus, we investigated whether the epacadostat-dependent induction of the tolerogenic phenotype in pDCs could be due to the signaling pathway activated by IDO1. To this purpose, we conditioned pDCs with the compound or the vehicle alone and we then analyzed the gene transcription of key molecules involved in the IDO1-mediated self-maintaining regulatory circuitry, namely *Ido1* and *Tgfb1* (**Figure 4B**). We did detect induction of the above genes after stimulation with epacadostat, confirming that the compound favors the activation of a positive immunoregulatory circuitry in pDCs (**Figure 5**).

## Discussion

Epacadostat (firstly named INCB024360) was one of the most promising catalytic inhibitors targeting IDO1 to foster anti-tumor immune responses. In fact, IDO1 has a role in establishing an immunosuppressive tumor microenvironment in many tumors through its enzymatic activity, which causes tryptophan depletion and the production of kynurenine and several metabolites (kynurenines). As a consequence of the IDO1 activity, it is impaired the proliferation of effector T cells as well as are inactivated T cells and natural killer (NK) cells (24, 25). Moreover, regulatory T cells (T_regs_), DCs and myeloid-derived suppressor cells (MDSCs) are induced and activated (26). A crucial role is played by the kynurenine, which can directly interact with AhR to mediate the activation of the receptor signaling and the regulation of the immune response (22). As a consequence, upregulation of IDO1 expression in tumor cells, programmed cell death 1 (PD-1) in CD8^+^ T cells and forkhead box P3 (FOXP3) in CD4^+^ T cells occur, overall impairing CD8^+^ T cells proliferation and promoting the conversion of CD4^+^ T cells to T_regs_ (27–29). Altogether, these events favor the formation of an immunosuppressive microenvironment that allows tumor growth. Hence, the finding that IDO1 is overexpressed in many tumors together with the delineation of its role in tumor immune resistance made IDO1 an attractive drug target in oncology.

Epacadostat was developed to meet these needs. It is a small molecule orally administered that competitively and selectively blocks the tryptophan-degrading activity of IDO1 but not of IDO2 (indoleamine 2,3-dioxygenase 2) or TDO (tryptophan 2,3-dioxygenase), two other tryptophan-degrading enzymes (8, 30). Being effective in many preclinical tumor models, it entered several clinical trials and promising results were observed in a phase I/II trial in combination with the anti-PD-1 pembrolizumab (31, 32). In any case, in most models the anti-tumor activity of epacadostat is best seen not as a single therapy, but in combination with other immunotherapy agents (33). However, the subsequent large phase III trial, named ECHO-301/KN-252, testing epacadostat in combination with pembrolizumab in advanced melanoma patients did not show any benefit in comparison to patients in therapy with the anti-PD-1 alone. As a consequence, this trial was stopped and many others were blocked or downsized (9). Several possible explanations for this negative result have been hypothesized (10), but many aspects have not yet been investigated.

It was identified that epacadostat potently activates AhR-mediated signaling, inducing the nuclear translocation of AhR and the upregulation of IDO1 expression (34) in both tumor cells and splenic T cells, leading to immunosuppression (35).

Recent and growing evidence describes the existence of an apo-form of IDO1 (namely, the protein without heme cofactor and thus catalytically inactive) in the tumor microenvironment that could contribute to the immunoregulatory milieu *via* its non-enzymatic activity (36–38).

In this study, we demonstrate that epacadostat can also affect the non-enzymatic function of IDO1. In the mastocytoma tumor cell line P1.HTR stably expressing the mouse IDO1 protein, epacadostat can effectively inhibit the enzymatic activity of IDO1 with an IC50 of 54,46 ± 11,18 nM. This value is quite similar to the one described in the literature for mouse IDO1 overexpressed in B16F10 melanoma (88 nM) (21), but higher than the one measured with the human protein (10 nM) (30). When used in P1.IDO1 cells, epacadostat favors the first events activating the IDO1-mediated signaling pathway, i.e. IDO1 binding to SHPs and also to the PI3K regulatory subunit p85, as well as the IDO1 anchoring to the EEs. These findings suggest that in tumor cells overexpressing IDO1, epacadostat could lead to the activation of a signaling pathway responsible for the induction of an immune suppressive phenotype. Our previous data demonstrated that the enzymatic and the non-enzymatic functions of IDO1 reside in different and mutually exclusive conformations (15, 16), we thus speculated that epacadostat could stabilize the non-enzymatic conformation of IDO1 that is more prone to interact with its partners SHPs and PI3K, at the expenses of its catalytic activity.

Moreover, our data reveal that in pDCs epacadostat induces the gene transcription of key molecules involved in the IDO1-mediated self-maintaining regulatory circuitry, namely *Ido1* and *Tgfb1*. As a consequence, epacadostat confers an *in vivo* detectable immunosuppressive phenotype in pDCs.

Overall, these data suggest that a possible explanation for the failure of epacadostat in the phase III trial ECHO-301 is a dual role of the compound. In fact, it has both the desired effect (i.e., blocking the catalytic activity of IDO1), but also an opposed undesired effect (i.e., enhancing its signaling activity). Growing evidence indicates the existence of a cell-specific and dynamic balance between the holo- and the apo-conformations of IDO1 that respectively mediate the enzymatic and non-enzymatic functions of the protein. The metabolic factors leading to the stabilization of an intracellular pool of either IDO1 conformations are still not fully characterized. The resulting effect deriving from the combination of the catalytic inhibition and the enhancement of the signaling function of IDO1 may strongly depend on the cell type (i.e., tumor and/or immune cell populating the tumor microenvironment). Moreover, the metabolic profile of the cell (i.e., heme availability, competition with other heme-proteins, redox status of iron), could also affect the shift between the two conformations of IDO1. Lastly, the expression of the IDO1 interactors in the cell may promote the signaling rather than the enzymatic function of IDO1. Further studies are required to deepen this new prospective on IDO1.

In conclusion, for the first time, this study shows that epacadostat, a small molecule developed as catalytic inhibitor of IDO1, may also promote the signaling function of IDO1 and induce an immune suppressive phenotype in pDCs. These data reinforce the idea that IDO1 targeting in the tumor microenvironment must take into account the ‘moonlighting’ activity of this immune checkpoint protein. Therefore, the inhibition of the non-enzymatic function of IDO1 could represent an alternative and promising approach in the anti-tumor immunotherapy.

## Data availability statement

The original contributions presented in the study are included in the article/**Supplementary Material**. Further inquiries can be directed to the corresponding author.

## Ethics statement

All animal studies were approved by the Italian Ministry of Health and done in compliance with national (Italian Parliament DL 116/92) and Perugia University Animal Care and Use Committee guidelines.

## Author contributions

MTP designed the study, supervised the experiments and wrote the paper; EP, GM, CS, SR, and MG performed *in vitro* experiments and analyzed the data; CV and GM performed *in vivo* experiments and analyzed the data; CO and MLB reviewed and edited the manuscript. All authors contributed to the article and approved the submitted version.
