# Supplementary material for: The catalytic inhibitor epacadostat can affect the non-enzymatic function of IDO1
Source: Front Immunol. 2023 Apr 14;14:1134551. doi: 10.3389/fimmu.2023.1134551 (PMC10145169; doi:10.3389/fimmu.2023.1134551)
Supplement: Supplementary file 1 [file DataSheet_1.pdf]

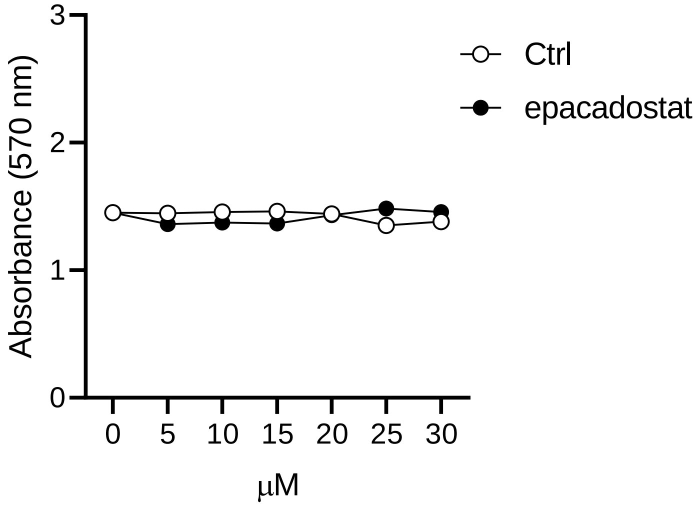

Supplementary fig. 1. Epacadostat does not affect viability of P1.HTR tumor cells. (A) Cell viability percentage (%) of P1.HTR cells treated with epacadostat, or the vehicle alone as a control (Ctrl). Results are the mean ± SD of three experiments, each conducted in triplicate.

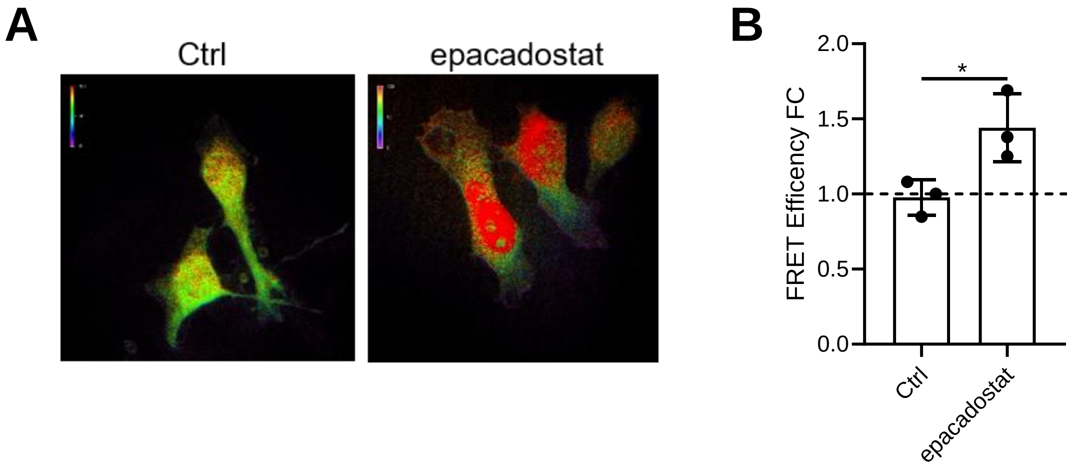

Supplementary fig. 2. Epacadostat favors the IDO1 binding to SHP-2. (A) Representative FRET microscopy images of GFP/RFP emission in HeLa cells expressing IDO1-RFP and SHP-2-GFP fusion protein treated with epacadostat (1µM) or the vehicle alone as a control (Ctrl). Pseudocolor scale, red corresponds to maximum interaction, while purple indicates the absence of proteins proximity. One experiment representative of two is shown. (B) FRET efficiency values (mean ± S.D.) are presented as fold change (FC) of cells treated with epacadostat relative to untreated counterparts (fold change = 1, dotted line). *$p < 0.05$

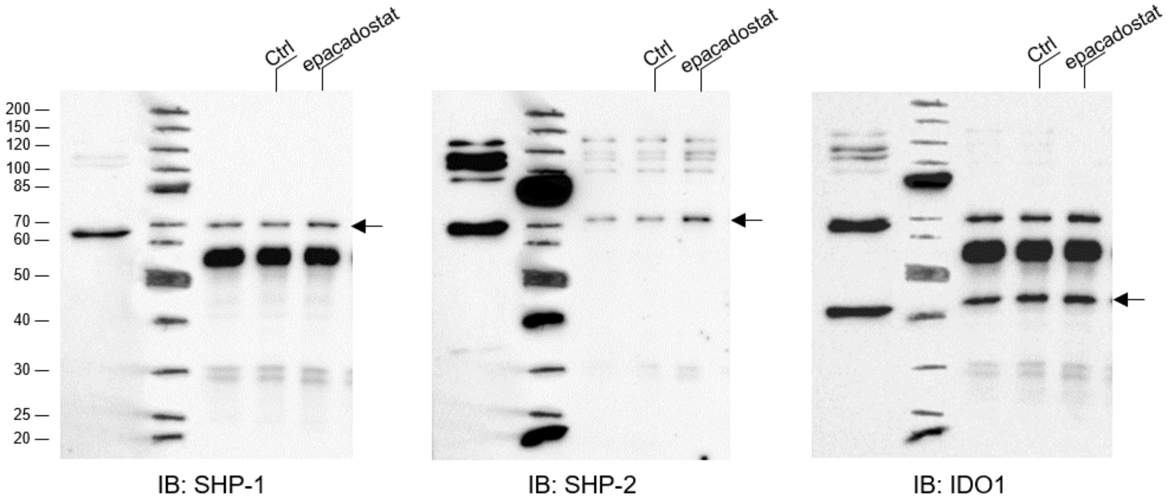

Supplementary fig. 3. Whole gels of the original Western blots shown in Fig. 2D.

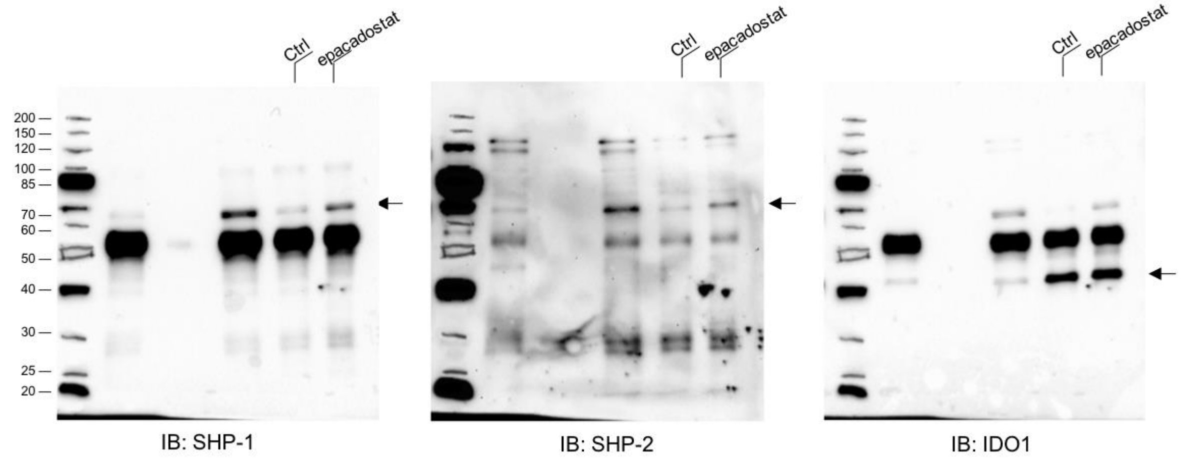

Supplementary fig. 4. Whole gels of the original Western blots shown in Fig. 2G.

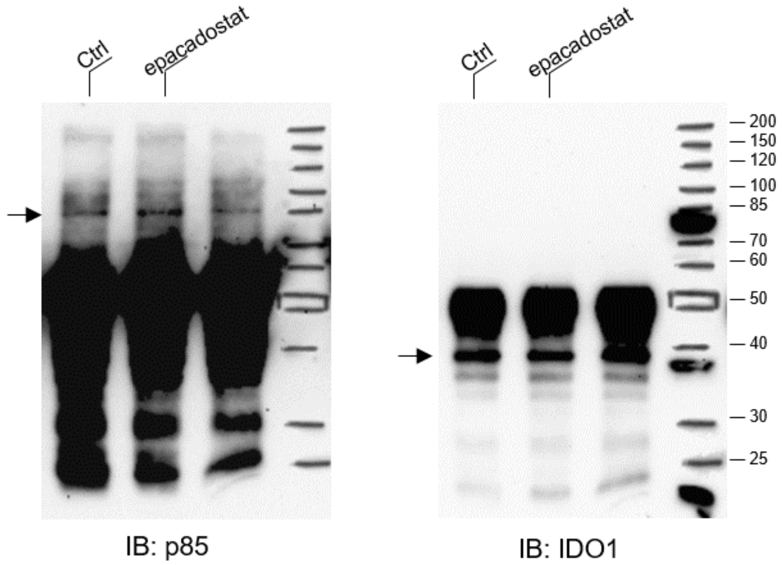

Supplementary fig. 5. Whole gels of the original Western blots shown in Fig. 3A.

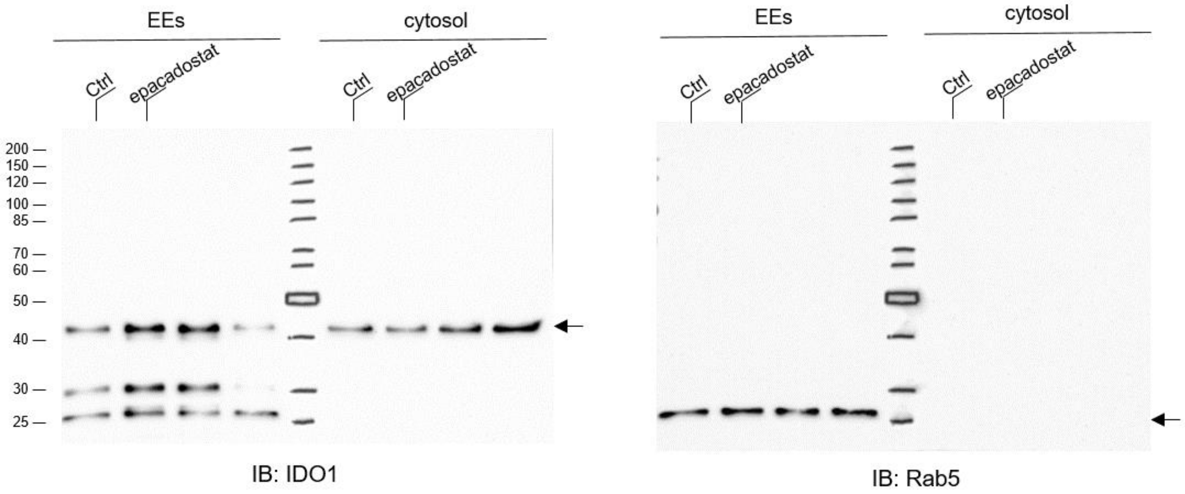

Supplementary fig. 6. Whole gels of the original Western blots shown in Fig. 3C.

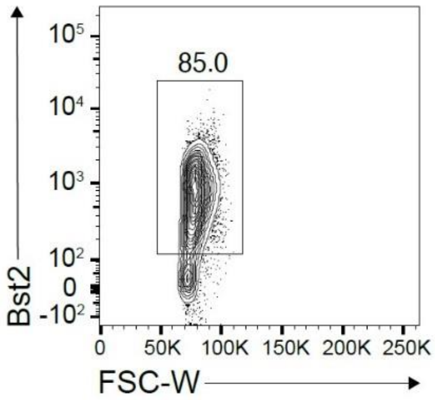

Supplementary fig. 7. Shown is the relative percentage of pDCs (Bst2$^+$) purity. One representative experiment (n =3).

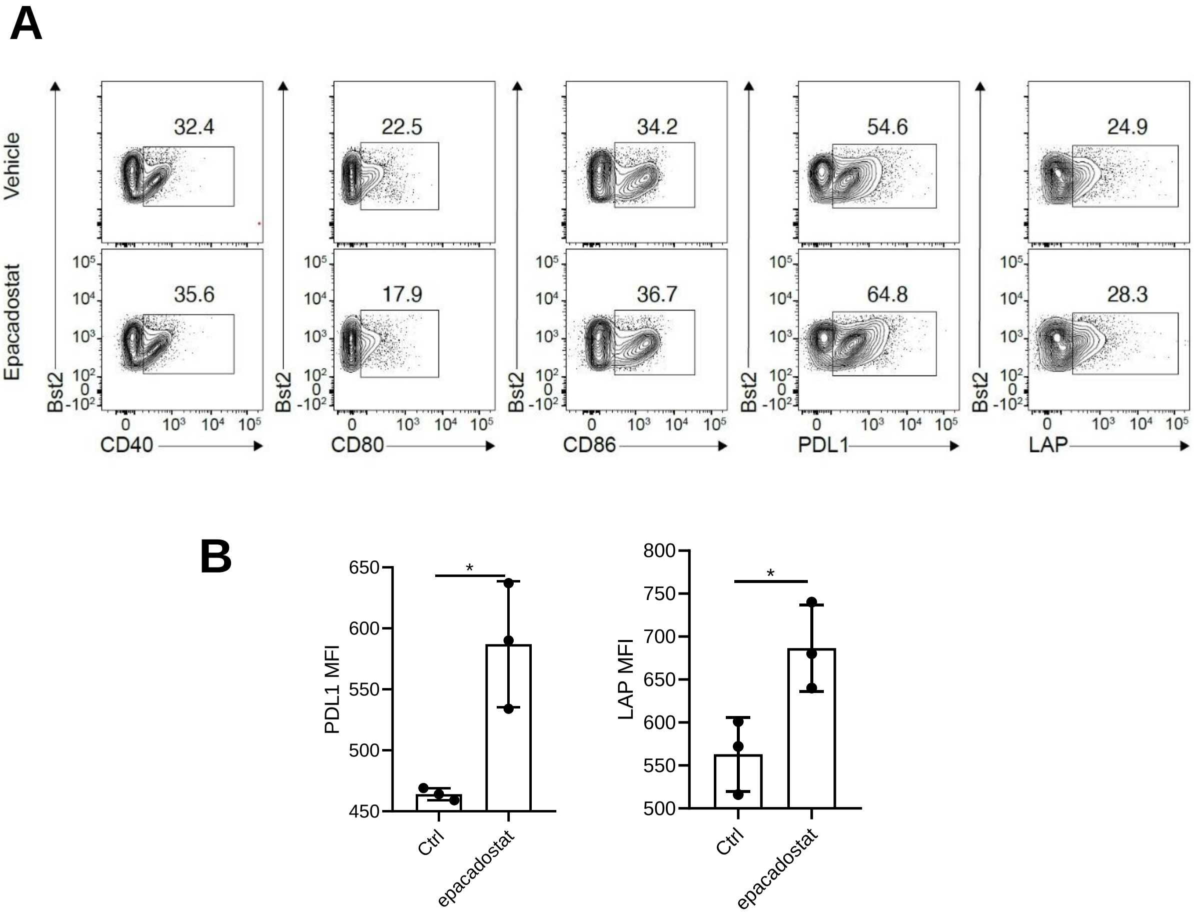

Supplementary fig. 8. Epacadostat affects the pDCs phenotype. (A) pDCs were treated with epacadostat or the vehicle alone as a control (Ctrl) and expression of CD40, CD80, CD86, PDL1, LAP was evaluated in cells pre-gated on Bst2 (n = 3). **(B)** Quantification of PDL1 and LAP expression (MFI) in pDCs gated on Bst2 (n = 3). Data are shown as mean ± SD. * $p < 0.05$.
